# Beyond Canonical Neoantigens: Emerging Technologies for Identification of Noncanonical Antigens and Implications for Personalized Cancer Vaccines

**DOI:** 10.3390/cancers18172779

**Published:** 2026-08-27

**Authors:** Yilin Yang, Thomas Kane, Abdurrahman T. Abdelzaher, S. Peter Goedegebuure, William E. Gillanders

**Affiliations:** 1 Department of Surgery, Washington University School of Medicine, Saint Louis, MO 63110, USA; yiliny@wustl.edu (Y.Y.); abdelzaher@wustl.edu (A.T.A.); goedegep@wustl.edu (S.P.G.); 2 School of Medicine, University of Missouri, Columbia, MO 65211, USA; 3 The Alvin J. Siteman Cancer Center, Washington University School of Medicine and Barnes-Jewish Hospital, Saint Louis, MO 63110, USA

**Keywords:** personalized cancer vaccines, noncanonical antigens, transposable elements, circular RNA, long noncoding RNA, defective ribosomal products, long-read sequencing, ribosome profiling, immunopeptidomics, proteogenomics

## Abstract

Over the past decade, advances in sequencing technologies and computational pipelines enabled the development of personalized cancer vaccines (PCVs). Current PCV strategies primarily target cancer neoantigens generated by non-synonymous DNA mutations, which can result in altered amino acid sequences capable of eliciting tumor-specific immune responses. More recently, a distinct class of tumor-specific antigens (TSA), termed noncanonical or cryptic antigens, has emerged as an additional source of immunogenic targets. Unlike canonical neoantigens, noncanonical antigens typically cannot be identified by tumor/normal whole-exome sequencing, as they do not arise from classical DNA mutations. Instead, they are often associated with less well recognized and/or aberrant processes in the pathways from DNA to human leukocyte antigen (HLA)-presented peptides. Examples include transposable elements, circular RNA, translation of alternative open reading frames and/or long non-coding RNA, among others. Emerging evidence suggests that noncanonical antigens represent a substantial portion of the tumor-specific immunopeptidome and, similar to canonical neoantigens, are absent during thymic selection and can evade central tolerance and elicit T cell responses. Technological advances have increasingly facilitated the identification of noncanonical antigens. Long-read RNA sequencing reveals noncanonical transcripts by improving transcriptome assembly, while ribosome profiling provides genome-wide maps of actively translated regions, facilitating the discovery of peptides from aberrant translation events. Specialized molecular approaches enable enrichment and sequencing of circular RNAs, and immunopeptidomics using mass spectrometry allows for direct characterization of HLA-presented peptides. Together, these technological advances have led to an increasing interest in prioritizing and targeting noncanonical antigens in the next generation of PCVs. This review provides an overview of the diverse origins of TSAs beyond classical neoantigens and discusses emerging approaches that may enable the integration of these antigens in future clinical trials.

## Simple Summary

Cancer cells often produce proteins not found in healthy cells. Personalized cancer vaccines (PCV) are designed to train the patient’s immune system to recognize these unique proteins and attack cancer cells while sparing normal tissues. Currently, PCV targets are usually derived from genetic mutations in a patient’s tumor identified on sequencing. However, growing evidence suggests that many cancer-specific proteins actually arise from aberrant processes in transcription and translation, even in the absence of DNA mutations. Compared to mutation-derived proteins, these proteins are often more structurally distinct and can be especially effective at triggering immune responses. New technologies, such as long-read DNA/RNA sequencing, circular RNA sequencing, ribosome profiling, and mass spectrometry, have made it possible to identify previously hidden cancer-specific proteins. In this review, we describe the diverse sources and identification technologies of aberrant cancer-associated proteins beyond genetic mutations and highlight their clinical relevance in next-generation PCVs.

## 1. Introduction

Personalized cancer vaccines (PCVs) have emerged as a promising immunotherapeutic strategy through the targeting of tumor-specific antigens (TSAs), i.e., antigens that are exclusively expressed in tumor cells. Over the past decade, advances in next-generation sequencing (NGS) and computational neoantigen prediction pipelines have enabled the development of PCVs targeting classical neoantigens, a subset of TSAs that usually arise from nonsynonymous single nucleotide variants (nsSNVs) and insertions/deletions (INDELs). To identify classical neoantigens, current PCV platforms primarily rely on matched tumor–normal whole-exome sequencing (WES) and tumor RNA sequencing (RNA-seq). Early clinical studies have demonstrated the feasibility, safety, and immunogenicity of this approach across multiple cancer types [1]. However, many tumors, particularly those with low tumor mutational burden, harbor only a small number of targetable classical neoantigens, limiting PCV design. Additionally, even in tumors with more targetable mutations, clinical responses to PCV remain variable [1].

Increasing evidence suggests that the TSA landscape extends far beyond classical mutation-derived neoantigens [2,3]. Collectively termed noncanonical antigens, these additional sources of TSAs arise from diverse genomic, transcriptomic, translational and post-translational processes such as transposable elements (TE), aberrant pre-mRNA splicing, translation of noncanonical open reading frames (ORFs) within circular RNA (circRNA) and long non-coding RNAs (lncRNA), proteasome-spliced peptides (PSP), and defective ribosomal products (DRiPs). Unlike classical neoantigens, noncanonical antigens often evade detection by conventional WES-based pipelines as they do not originate from classical coding region mutations. Nevertheless, many noncanonical antigens are absent during thymic selection and can evade central tolerance and elicit robust T cell responses. Some noncanonical antigens can also be shared among patients with the same cancer type [4] and across cancer types [5,6], which could potentially drive the development of off-the-shelf cancer immunotherapy. Emerging studies further suggest that noncanonical antigens constitute a substantial fraction of the tumor immunopeptidome [2,3], highlighting their potential as an underappreciated source of immunotherapeutic targets.

Recent technological advances are progressively enabling the discovery of noncanonical antigens. Long-read RNA sequencing and ribosome profiling (Ribo-seq) are improving the detection of noncanonical transcripts and translation events, while specialized molecular and computational workflows have been developed to identify circRNA-derived antigens. In parallel, mass spectrometry (MS) now permits direct characterization of peptides presented by human leukocyte antigen (HLA) molecules. In fact, proteogenomics—a field aiming to integrate advanced sequencing and immunopeptidomic data—has made strides in systematic and comprehensive TSA identification. In this review, we provide an overview of the current landscape of canonical and noncanonical antigens, discuss emerging technologies used for their discovery and predicted immunogenicity, and highlight the growing clinical relevance of expanding the antigenic repertoire for next-generation PCVs.

## 2. Classical Neoantigens

Classical neoantigens are defined as tumor-specific peptides derived from mutational events in protein-coding regions of the genome that can potentially elicit a tumor-specific immune response. This principle has laid the foundation for PCVs, which aim to amplify antitumor T-cell responses against patient-specific tumor mutations. Since the first published neoantigen vaccine trial in 2015 [7], classical neoantigens have informed 100+ personalized neoantigen vaccine clinical trials [8]. Broadly labeled classical neoantigens can be subdivided into three categories based on the mutational event: nsSNVs, INDELs, and gene fusions.

### 2.1. Nonsynonymous Single Nucleotide Variants (nsSNVs)

nsSNVs are the largest source of classical neoantigens, comprising up to 95% of somatic coding mutations across solid tumors from The Cancer Genome Atlas (TCGA) [9]. Biochemically, nsSNV-derived neoantigens arise from a single nucleotide base pair alteration that precipitates an amino acid substitution in an encoded protein. nsSNV neoantigen-HLA complexes can elicit an antitumor immune response, although their structural similarity to the wild-type peptide-HLA complex can reduce immunogenicity relative to neoantigens originating from other, more disruptive mutational events (e.g., gene fusions, frameshift INDELs) [9]. The efficacy of nsSNV-derived neoantigens is therefore highly reliant on the position and biochemical attributes of the substituted amino acid residue. For example, non-anchor nsSNVs alter the T cell receptor (TCR)-facing surface of the peptide-HLA complex and therefore require sufficient structural dissimilarity from self to escape immune tolerance. In contrast, anchor nsSNVs alter residues that mediate peptide binding within the HLA groove, potentially enabling the presentation of a peptide that was previously undisplayed, creating a novel epitope to which the immune system has not been tolerized [10]. Although anchor nsSNVs are less numerous than their non-anchor counterparts, they may be more likely to potentiate high-quality tumor-specific immune responses [11]. Overall, nsSNVs have constituted the dominant candidate pool for PCV design due to their general abundance across cancer subtypes and their straightforward identification using tumor-normal sequencing [9,12].

### 2.2. Insertions/Deletions (INDELs)

Classical INDEL neoantigens are generated by insertion or deletion of one or more nucleotides within a protein-coding region of DNA. When these alterations are not divisible by three, they potentiate a frameshift, contributing a novel downstream amino acid sequence absent from the normal proteome. Compared to nsSNVs, INDEL neoantigens are less subject to central tolerance due to their larger deviation from self peptides. INDELs are especially relevant in microsatellite instability (MSI)-high tumors or tumors lacking appropriate mismatch repair, most prevalent among endometrial and colorectal cancers (CRC) [13,14], likely related to enzyme infidelity as the DNA polymerase “slips off” tandem MSI sequences [15]. In a pan-cancer analysis, Turajlic et al. found that INDEL-derived neoantigens, while substantially less prevalent than nsSNVs, generated approximately a threefold higher affinity HLA binding and approximately a ninefold more mutant-allele-specific binders compared to nsSNV-derived neoantigens, suggesting frameshift INDELs generate peptides that are both efficiently presented and more distinct from self [9]. These findings reinforce the importance of neoantigen prioritization in vaccine design.

### 2.3. Fusion Genes

Despite being DNA-level classical neoantigens in the strictest sense, fusion events are challenging to identify by WES, particularly when they involve intronic regions. Even with high sequencing depth, the majority of fusion genes detectable by WES are located within or near captured exons [16]. In contrast, RNA-seq (with special fusion-detection algorithms) has a higher sensitivity in detecting fusion events, as it can avoid the need to sequence through large introns. In fact, current clinical guidelines from NCCN and ASCO recommend RNA-seq as the preferred method for detecting gene fusions [17].

Well-known examples of gene fusion-derived PCV targets in the clinical trial setting include the *BCR-ABL* fusion in chronic myeloid leukemia [18,19] and the *DNAJB1-PRKACA* fusion in fibrolamellar hepatocellular carcinoma [20,21,22]. Gene fusions may precipitate neoantigens that are potentially more immunogenic than those derived from nsSNVs or INDELs, because each fusion event can generate multiple targetable neoepitopes without a corresponding wild type. Indeed, Weber et al. reported that gene fusion-derived antigens demonstrated an immunogenicity rate of 48%, significantly exceeding the 19% rate observed for nsSNVs [23]. Targeting gene fusion-derived neoantigens may also affect tumor development since they are frequently driver mutations [24].

## 3. Methodology for Identifying Classical Neoantigens

PCVs targeting classical neoantigens rely on genetic sequencing and bioinformatic algorithms to predict HLA-presented neoantigens. The classic approach is via matched tumor-normal WES and tumor RNA-seq. After WES data is mapped to a reference genome, sequencing variants are identified through variant caller algorithms [25,26,27,28,29,30,31]. Sequencing data from matched normal tissue—usually from peripheral blood mononuclear cells (PBMCs) in clinical workflows—is also incorporated to filter out patient-specific germline variants.

While WES informs what mutations are present, RNA-seq sheds light on mutation expression and abundance. This allows for validation of neoantigens identified by WES and prioritization of neoantigens that are dominantly expressed [32,33,34]. While most frequently used as an expression-based filtering tool for DNA-derived variants, recent work has demonstrated that RNA-seq data alone can identify variants and enable neoantigen prediction independent of WES [35]. Incorporating RNA-seq data into neoantigen prediction has thus become the gold standard for clinical cancer vaccine studies. Several software platforms, such as pVACtools [36] and TSNAD [37], integrate both WES and RNA-seq data in the analysis algorithms, enhancing the specificity of neoantigen identification.

**Figure 1:**
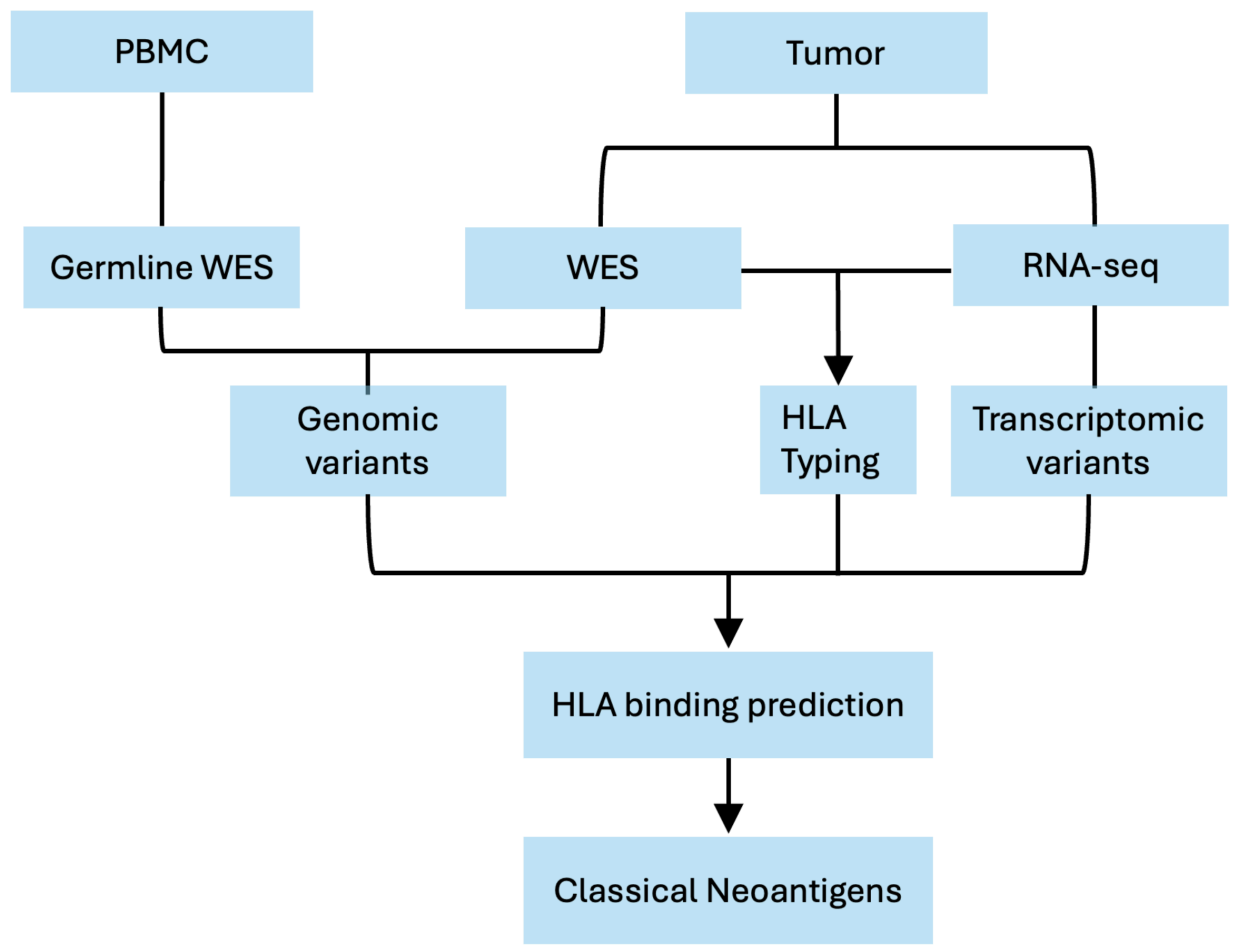
Current classical neoantigen prediction workflow utilizing whole exome sequencing and RNA sequencing. PBMC: peripheral blood mononuclear cells; WES: whole-exome sequencing; RNA-seq: RNA sequencing; HLA: human leukocyte antigen.

In a cancer neoantigen prediction workflow, a patient’s HLA type is then determined by WES data and/or RNA-seq data. Figure 1 highlights the key steps in the most widely used current approach to identify mutation-derived classical neoantigens.

Many clinical studies to date have employed diverse PCV platforms, including mRNA, DNA, peptide, dendritic cells, and viral vectors, taking advantage of each platform’s unique strength [38,39]. Furthermore, as cancers frequently exhibit tumor heterogeneity, restricted antigen presentation, immune evasion, and an immunosuppressive microenvironment, the synergistic potential of combining PCV with other cancer immunotherapies has been increasingly recognized and adopted in clinical practice [40]. Overall, accumulating clinical trial evidence [41,42] suggests that classical neoantigen PCVs remain a highly promising approach in cancer immunotherapy, particularly as vaccine platforms and combination strategies continue to rapidly evolve.

## 4. Noncanonical Antigens: Tumor-Specific Antigens Beyond Classical Neoantigens and Their Importance

The contemporary PCV design approach integrating WES and RNA-seq data limits the discovery of TSAs to only the DNA-level mutations located in the exonic coding region and systematically excludes multiple additional classes of TSAs (Table 1). Collectively termed “cryptic”, or noncanonical antigens, these additional TSA classes do not arise from canonical DNA mutations and therefore can escape detection on tumor versus normal tissue WES. Instead, they are often associated with less well-recognized processes in transcription and translation. Advances in proteomic technologies and their application in cancer immunology research have led to the discovery that noncanonical antigens represent a substantial portion, if not the majority, of the tumor-specific immunopeptidome. For example, a 2018 study on both murine cell lines and human tumors showed that 90% of TSAs were derived from allegedly noncoding sequences [2]. Another more recent study showed that only 1% of TSAs are derived from mutated sequences in human melanoma and lung cancer [3]. Importantly, similar to classical neoantigens, noncanonical antigens are absent during thymic selection and, when presented by HLA molecules, can evade central tolerance to elicit downstream T cell responses.

**Figure 2:**
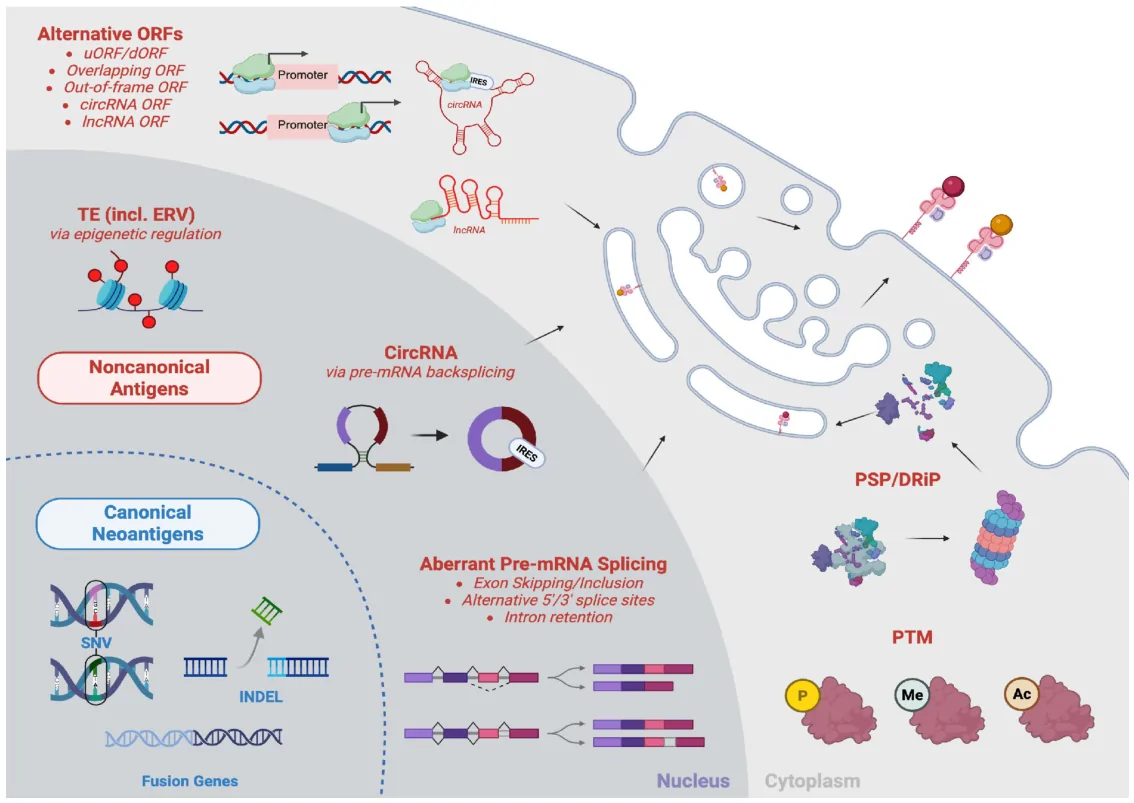
Sources of tumor-specific antigens at multiple stages from the genome to human leukocyte antigen (HLA)-presented peptides. Arrows represent the intracellular pathway for HLA presentation: peptide synthesis → peptide loading in the endoplasmic reticulum → Golgi apparatus → plasma membrane.

Numerous sources of noncanonical antigens have been identified across multiple cancer types (Table 1 and Figure 2). Aberrant processes generating these antigens can occur at multiple stages from the genome to HLA-presented peptides, including genomic/epigenomic, transcriptomic, translational, proteomic, and antigen processing pathways, with some of the pathways overlapping with and complementing one another. Shared mechanisms can exist between canonical and noncanonical pathways as well. For example, enzymatic slippage that produced canonical INDEL neoantigens could also occur at the transcriptional level, producing RNA frameshift antigens [43]. Noncanonical antigens have great potential to expand the TSA repertoire in PCV design. In this section, we discuss common sources of noncanonical antigens and their involvement in cancer.

### 4.1. Transposable Elements (TE) and Endogenous Retroviruses (ERVs)

One class of DNA-level, noncanonical antigen is TE. TEs are repetitive sequences and can comprise up to half of the human genome [44]. Far from being “junk DNA,” TEs have been exapted for essential biological functions such as genome regulation [45] and innate immunity [46]. While the majority of TEs are silenced epigenetically, epigenetic dysregulation in cancer can lead to TE reactivation and genome instability [47]. Reactivated TEs, such as Alu, long interspersed nuclear element-1 (LINE1), and endogenous retrovirus (ERV)-derived sequences, can transpose within the genome and insert themselves into both coding regions of oncogenes and tumor suppressor genes and important regulatory elements such as promoters and/or alternative splicing sites [48]. Such disruption has been implicated in the carcinogenesis of a multitude of cancer types, including but not limited to brain, head and neck, breast, lung, and colorectal [44,49].

More recently, research has shed light on the potential of targeting TEs in various cancers. In addition to their regulatory roles in carcinogenesis, TEs have been shown to be translated into novel proteins, making them a source of noncanonical antigens [50]. For instance, both LINE1 and ERV elements contain ORFs and can directly encode native TE proteins that are tumor-specific [51,52,53]. Additionally, when transposed into other coding transcripts, TEs can generate chimeric proteins, some of which encode immunogenic T cell peptides and can serve as PCV targets in multiple cancer types [6,54,55]. Importantly, TE transcripts have been shown to produce “danger signals” for the immune system, such as double-stranded RNA, and create a viral mimicry state [56] in cancer cells. The viral mimicry state created by TE expression may prime a cancer to be more susceptible to immune responses. Several trials are underway to explore the therapeutic value of combined treatment with an epigenetic drug and immune checkpoint blockade (NCT03220477, NCT03903458, NCT0440774), leveraging the activated innate immune system for optimized response to immune therapies. Whether PCVs containing TE-derived TSAs could generate antitumor immune responses warrants further investigation. Reassuringly, recent pre-clinical data using PCVs containing TE-derived TSAs combined with anti-PD-1/PD-L1 or IL-15 superagonist N-803 showed expansion of TE-specific T cells and tumor control in murine models [57].

### 4.2. Aberrant Splicing Events and Circular RNA (circRNA)

Aberrant transcriptional events such as dysregulated pre-mRNA splicing (exon skipping, intron retention, and alternative 5′ or 3′ splice site usage) produce noncanonical antigens. They are prevalent in cancer and have been reviewed in depth [58,59]. The relative proportion of splicing-derived noncanonical antigens far exceeds that of classical neoantigens—in one study on hepatocellular carcinoma (HCC), aberrant splicing-derived TSAs accounted for more than half of all TSAs compared to only 4.4% for those derived from coding region mutations [60]. Preclinical proof-of-concept studies have demonstrated the efficacy of PCVs encoding aberrant splicing-derived TSAs, resulting in significant tumor regression and enhanced intratumoral infiltration of neoantigen-reactive T cells in HCC mouse models [60].

A subset of aberrant splicing events—back splicing—creates circRNA. Formed by covalent joining of a downstream 5′ splice donor site to an upstream 3′ splice acceptor site, forming a stable, circular structure [61], circRNA has been demonstrated to possess multifaceted roles in cancer. For instance, they are implicated in cancer progression by microRNA sponging, competitive protein binding leading to dysregulation of cancer-associated biological pathways, and modulation of the tumor microenvironment [62]. Notably, emerging evidence suggests that circRNAs can be translated into proteins, challenging the long-held belief that they are strictly non-coding. CircRNA translation can be 5′ cap-dependent [63], where the new backsplice junction (BSJ) can lead to out-of-frame, novel translational products. More importantly, circRNA translation can also initiate internally at internal ribosomal entry sites (IRESs) [64,65], generating out-of-frame peptides. These processes generate a broad repertoire of noncanonical antigens in the tumor context.

CircRNA-derived noncanonical antigens have increasingly been identified and explored as targets for PCV design. An important study by Huang et al. in 2024 [66] demonstrated that a breast tumor-specific circRNA, circFAM53B, undergoes noncanonical translation to produce peptides that strongly bind both HLA class I (HLA-I) and class II (HLA-II) molecules, functioning as bona fide noncanonical antigens. Around the same time, circRNA-derived peptides have been identified by MS in microsatellite-stable CRC [67], lung cancer, and melanoma [68]. These results attest to the distinct circRNA landscape that cancers possess and further support the rationale to exploit circRNA as a source of noncanonical antigens in PCV design.

Similar to other noncanonical antigens, circRNA-derived TSAs may be more immunogenic than classical neoantigens because the noncanonical translation produces peptides that are entirely absent from the canonical proteome and are thus more likely to escape central tolerance [69]. In one of the few studies to date, tumor-bearing mice vaccinated with circRNA-derived TSA showed enhanced infiltration of circRNA antigen-specific cytotoxic T cells and effective tumor growth control [66]. These early, promising results call for additional preclinical and clinical vaccination studies with circRNA-derived noncanonical antigens.

### 4.3. Long Noncoding RNA (lncRNA) and the Noncanonical Open Reading Frames (ORFs)

Advances in proteomic and bioinformatic techniques have led to the discovery of dysregulated processes in translation mediated by noncanonical ORFs: regions of translation that fall outside the traditional protein-coding annotation [70]. Examples of noncanonical ORFs include but are not limited to upstream ORFs (uORFs), downstream ORFs (dORFs), overlapping and/or out-of-frame uORFs/dORFs, ORFs within lncRNAs, circRNA ORFs, and pseudogenic ORFs. Microproteins encoded by noncanonical ORFs have been shown to account for 40% of TSAs in HCC, with several exhibiting immunogenicity in humanized mice [71]. Multiple reviews [70,72,73] have defined and described in detail the relevance of these TSA categories in the context of cancer.

Within noncanonical ORF categories, lncRNA-derived peptides represent a rapidly emerging class of noncanonical antigens with significant potential for PCV development. These allegedly “non-coding” sequences have been shown to contain small ORFs that are translated into short peptides [74,75] and recognized as a major untapped reservoir of immunogenic PCV targets. For instance, MS analysis of normal and CRC tissues has identified a peptide encoded by lncRNA PVT1 in multiple CRC samples [76]. Furthermore, PVT1 was recognized by both tumor-infiltrating lymphocytes and peripheral blood mononuclear cells, suggesting immune recognition and surveillance. Similarly, other studies have identified lncRNA-derived TSA enriched in melanoma [77] and HCC [71] samples. Notably, researchers have advocated renaming “long noncoding RNA” to “long noncanonical RNA”, given the increasing recognition of their translation potential and clinical relevance [74].

The relevance of lncRNA in cancer, as well as the immunogenicity of lncRNA-derived TSAs, is best exemplified through a study by Barczak et al. in 2023 [78]. The authors showed that in CRC cells, inhibiting protein arginine methyltransferase 5 (PRMT5), an enzyme that regulates the non-coding genome, altered the differential expression of lncRNA and caused a significant delay in tumor growth in tumor-bearing mice [78]. This is in line with the knowledge that lncRNAs play crucial roles in tumorigenesis and metastasis [79] and also serve as modulators of the tumor microenvironment [80,81]. In the same study, peptide PCVs containing lncRNA-derived TSAs led to increased CD8+ lymphocyte infiltration and delayed tumor growth in animal models [78]. These results strengthen the rationale for exploring lncRNA-derived TSAs as a class of epitopes in PCVs.

### 4.4. Post-Translational Modification (PTM) and Proteasome-Spliced Peptides (PSP)

After translation, peptide products can be further modified by cellular machinery, generating altered epitopes. The most well-known pathway is through post-translational modification (PTM), which includes processes such as acetylation, oxidation, citrullination, phosphorylation, and others. PTMs can vastly reshape the tumor antigenic landscape. For instance, a study in 2023 showed that PTMs generated thousands of TSAs on HLA-I molecules that are shared between patients and across cancer types but absent from normal tissue [82]. The underlying reason for the profoundly dysregulated PTM patterns in cancer cells is likely driven by factors including oncogenic signaling, metabolic reprogramming, and tumor microenvironmental stress [83]. PTM-derived TSAs, therefore, represent an emerging and promising class of PCV targets. A growing body of research is investigating the immunogenicity of PTM-derived TSAs, particularly those arising from citrullination [84], homocitrullination [85], and phosphorylation [83].

A less investigated source of post-translational, noncanonical antigens is proteasome-spliced peptides (PSPs). As the major protease hub responsible for generating antigenic peptides for CD8+ T cell recognition, the proteasome was originally thought to only produce 8–10 mers from linear, contiguous components of a parental protein. However, it is now recognized that it can also produce a subset consisting of spliced, chimeric sequences from two distant fragments in the parental protein [86]. PSP-derived TSAs have been reported in several cancer types, including melanoma [87,88], renal cell carcinoma [89], and CRC [90], supporting PSP-derived TSAs as a potential source of targetable noncanonical antigens in PCV design.

Evidence suggests that PSP-derived TSAs can be immunogenic. For example, Ebstein et al. (2016) showed that gp100-derived PSP epitopes activated CD8+ T cells from peripheral blood of ~50% of melanoma patients tested [88]. To date, however, there has not been any preclinical or clinical study using a PCV containing PSP-derived TSAs identified in tumor tissue that has successfully demonstrated an immune response. At the same time, controversy exists with regard to PSP’s frequency and immune relevance: a 2016 study reported that PSP-derived peptides account for approximately one-third of the HLA-I immunopeptidome [91]. However, a contrasting analysis in 2022 compiled three independent datasets of confirmed T cell antigens from cancer patients and found that only 1.4–7.2% of immunogenic antigens were PSP-derived, arguing for a marginal contribution to the pool of targetable TSAs [92]. To date, only eight PSP-derived TSAs have been validated [90,93]. This reflects a fundamental challenge to reliably identify bona fide, endogenous, PSP-derived TSAs. Some mass spectrometry specialists argue that many reported PSP-derived peptides may even represent artifacts [94]. Thus, whether the vaccine-targetable pool of PSP-derived TSAs can further expand with continued advances in proteomic techniques remains to be uncovered.

### 4.5. Defective Ribosomal Products (DRiPs)

First proposed by Yewdell et al. in 1996, the DRiP hypothesis posits that a significant fraction of major histocompatibility complex (MHC) class I peptide ligands derive from newly synthesized polypeptides that are rapidly degraded due to premature translation termination, misfolding, or failure to assemble into multisubunit complexes [95]. As originally described, these DRiPs are products of “translation of bona fide mRNAs in the proper reading frame” [95] and are therefore arguably “canonical” antigens. Yet, these short-lived, aberrant protein products remain largely invisible to the traditional WES and RNA-seq workflow (Figure 1), representing a new direction in PCV design.

Because DRiP-derived antigens are rapidly degraded by the proteasome, this generates a constant stream of peptides in proximity to the endoplasmic reticulum, where HLA-I peptide binding occurs. Thus, DRiPs can exhibit efficient HLA-I presentation and make up a large proportion of the HLA immunopeptidome [96]. Cancer cells, in particular, may exhibit increased ribosome translational infidelity compared to normal cells, possibly as a survival advantage [97,98], generating PCV-targetable DRiPs. For example, in pancreatic adenocarcinoma (PDAC), Zhang et al. identified single amino acid substitution peptides not attributable to genomic mutations or RNA editing, but rather to ribosomal translational errors. Rather than occurring stochastically, these errors are frequently due to tRNA-amino acid mis-acylation, where a wrong amino acid is attached to the tRNA due to chemical or structural similarities. Indeed, in this study, variant peptides were shared in multiple PDAC patients, making them good candidates as vaccine targets. Notably, these peptides can also be more immunogenic than their wild-type counterparts [99]. In another study, Champagne et al. also demonstrated that interferon-γ (IFNγ)-induced tryptophan depletion can cause cancer cells to produce DRiPs with tryptophan-to-phenylalanine (W > F) substitution via ribosomal slippage/frameshifting. Furthermore, T cells with high-affinity TCRs targeting the most broadly expressed W > F DRiP selectively killed tryptophan-depleted cancer cells but not normal cells [100]. These findings support the rationale to incorporate DRiP-derived TSAs in the design of next-generation PCVs.

### 4.6. Short-Live Proteins (SLiPs): A Broader Definition of DRiPs and Its Relevance to the Breadth of Noncanonical Antigens

Over time, peptides beyond the original DRiP definition [95] have been discovered to possess DRiP-like properties. Accordingly, the term “DRiP” has evolved to describe rapidly degrading polypeptides regardless of their origin [101]. In fact, researchers have proposed using the term “short-lived proteins” (SLiPs) to define the broad repertoire of DRiP-like peptides: antigens that are rapidly degraded and channeled to MHC-I molecules for presentation [102]. Many sources of noncanonical antigens behave like SLiPs. For instance, TSAs from noncanonical ORFs have been shown to generate HLA-I peptides 5-fold more efficiently per translation event than canonical protein-derived TSAs, precisely because they are inherently unstable and rapidly degraded, functionally resembling DRiPs [103]. IRES-dependent circRNA translation events generate proteins much shorter than mRNA-encoded proteins [104], which can be subjected to rapid proteasomal degradation in the cytoplasm [105] and, hence, more frequent HLA-I presentation. Similarly, aberrant pre-mRNA splicing events in cancer cells can produce premature termination codons and truncated proteins, which are rapidly degraded and directly channeled onto HLA-I [106]. Multiple recent studies have confirmed that peptides generated from aberrant pre-mRNA splicing are naturally presented by tumor HLA-I molecules and can elicit tumor-specific T cell responses [107,108].

SLiPs derived from noncanonical antigens might represent attractive PCV targets [102]. In addition to efficient HLA presentation, many of them also have the potential to be highly immunogenic because they are absent during thymic selection, and some are shared across tumor types [5]. However, little is currently known about HLA-II presentation of SLiPs. Since HLA-II preferentially presents the long-lived forms of a protein [109], the rapid degradation of SLiPs might preclude their presentation by class II molecules. Nevertheless, with the diverse sources and immunogenicity of noncanonical antigens, systematic discovery of noncanonical antigens has profound implications in developing the next generation of PCVs with enhanced efficacy.

## 5. Technologies for Noncanonical Antigen Identification

Methodological and bioinformatic advances have enabled identification of the different classes of TSAs. Here, we break down current methodologies in neoantigen identification into two categories: NGS-based in silico prediction and MS-based empirical data. Each methodology has its unique features and advantages/disadvantages in identifying specific classes of TSAs, and the methodologies are often used in combination. In addition to discussing the two types of technologies, we also review the proteogenomics pipeline: a field that systematically integrates genomic, transcriptomic, and proteomic data to enable more sensitive identification of canonical and noncanonical antigens.

### 5.1. Next-Generation Sequencing (NGS): Nucleotide Sequence-Based Antigen Discovery

#### 5.1.1. Long-Read Sequencing

Long-read sequencing, also known as “third-generation sequencing” [110], was developed around two decades ago to overcome the limitations of Illumina-based, next-generation short-read sequencing. Compared to short-read sequencing, which generates 50–300 bp sequence fragments for alignment and assembly, long-read sequencing can read up to tens of thousands of base pairs as one intact DNA or RNA fragment. This allows for de novo, continuous sequence assembly with fewer gaps and enables cell genomic and transcriptomic representation with higher resolution and accuracy.

There are currently two long-read sequencing platforms. PacBio (Pacific Biosciences) [111] uses illumination to watch a polymerase synthesize DNA from a DNA or RNA template in real-time, a technology termed “single-molecule real-time”, or SMRT. The second platform, ONT (Oxford Nanopore Technologies) [112], utilizes an electro-based base detection method to scan the DNA or RNA molecule as it passes through a nanopore. Because the base-calling algorithms rely on optical or electrical signals rather than traditional nucleotide fluorescent signals, both technologies were initially much more error-prone than Sanger or Illumina sequencing [113]. Over the years, however, both hardware and software updates have been implemented to improve the base-calling accuracy to close to 99.9% (Phred score Q30) for both platforms [114,115], nearing the accuracy achieved by Illumina sequencing.

The higher transcriptome resolution with long-read sequencing has proven to be a valuable tool in identifying multiple sources of noncanonical antigens. For example, long-read DNA sequences can span across genomic regions with high repetitiveness and enable high-resolution and accurate detection of TE-derived TSAs [116,117,118]. In parallel, long-read RNA sequencing effectively characterizes transcribed but unannotated sequences such as lncRNA [119], and those that harbor short similarity segments to the canonical transcriptome such as circRNA [120,121]. The length of sequenced molecules also becomes crucial in identifying aberrantly spliced RNA isoforms [122] and fusion genes [123,124,125].

Current limitations of long-read sequencing include cost and higher input DNA/RNA quantity and quality requirements, both of which may pose practicality challenges in clinical settings when resource material is limited. Clinical samples are often also preserved by formalin fixation and stored at room temperature for lower cost and ease of storage. This can lead to nucleic acid degradation and fragmentation [126], which can limit these samples’ compatibility with the long-read platform, which requires intact, high-molecular-weight molecules. In fact, the RNA integrity number (RIN), measured by capillary gel electrophoresis and calculated based on the relative intensity and ratio of the ribosomal 28S and 18S peaks, is an important quality control metric for input RNA for long-read sequencing. A high RIN (usually above 7) is required for successful long-read RNA library preparation. An additional limitation is that poly(A) selection is frequently utilized for long-read RNA-seq library enrichment. This may introduce 3′ end bias, skew the abundance metrics toward molecules with longer poly(A) tails, and miss any noncanonical transcripts that lack a poly(A) tail. Nevertheless, technical improvements are underway to expand the compatibility of the long-read platform with a broader range of clinical samples [127].

#### 5.1.2. Circular RNA Profiling

To sequence circRNA species, which are less abundant than mRNA in the cell, one first needs to enrich their relative proportion compared to mRNAs. One of the earlier enrichment strategies is using RNAse R transcriptome sequencing. By digesting the sample with an RNA exonuclease, linear mRNAs are selectively degraded [128], increasing the proportion of circRNA. Although this maximizes the signal-to-noise ratio, this method precludes concomitant profiling of mRNA and can be problematic if one is interested in the circRNA and mRNA isoform abundance ratio. Alternative methods include Ribo-Zero transcriptome sequencing [129] or exome capture sequencing [61], both of which deplete ribosomal RNA and, at the same time, preserve the mRNA-to-circRNA abundance ratio. Between the two methods, exome capture sequencing appears to be able to detect more circRNA species than Ribo-Zero in the same cell line or tissue [61]. In fact, one of the most comprehensive cancer-specific circRNA databases to date, MiOncoCirc [130], was generated using exome capture sequencing. More recently, Induro-RT-mediated circRNA sequencing (IMCR-seq) [131] was developed. By using a reverse transcriptase with rolling circle transcription activity followed by size selection, IMCR-seq can detect 6–1000 times more circRNAs for the benchmarked human samples compared to other methods, with as little as 10 ng of total RNA. This greatly increases the clinical applicability of circRNA profiling.

CircRNA sequencing data analysis methods can be broadly divided into two categories: those that focus on the chimeric BSJ-derived proteins and those that reconstruct the entire length of the circRNA isoform. To identify chimeric reads containing BSJs, only traditional short-read data is necessary, whereas to map and construct full-length isoforms, long-read sequencing is usually employed. Computational tools have been developed for both workflows. For short-read analysis, platforms like CIRI2 [132], CIRI3 [133], and KNIFE [134] can effectively capture the BSJ. Full circRNA isoform reconstruction with short-read data is also possible with tools like CYCLeR [135]. Nevertheless, with advances in both library preparations using rolling circle transcription such as IMCR-seq and long-read sequencing technologies, software tools such as CIRI-long [136] and isoCirc [121] have been developed to reconstruct complete circRNA isoforms with much higher resolution. More recently, a novel pipeline, CHRIS [137], was developed, integrating both short- and long-read RNA-seq data. In the benchmarking study using CRC samples, CHRIS enabled detection of many more novel circRNA species than either approach alone, in addition to better characterizing the distinct back-splicing events in each circRNA. These developments highlight the emerging role of circRNAs in cancer research and pave the path to better characterize cancer-associated circRNAs for PCV development.

#### 5.1.3. Ribosome Profiling (Ribo-Seq)

Ribo-seq, a relatively newer method in the NGS toolbox, enables high-throughput sequencing of only the mRNA molecules that are protected by ribosomes. This generates a library of transcripts about 28–30 nucleotides in length, known as the “ribosome footprints”, thereby providing a snapshot of regions of the transcriptome that are being actively translated [138]. Ribo-seq sample preparation generally includes halting translation and cell lysis, followed by nuclease digestion to generate ribosome-protected fragments and size-selection-based extraction of ribosome footprints from rRNA [139]. Extracted ribosomal footprints are then amplified into sequencing libraries either by sequential ligation of 3′ and 5′ adaptors to footprint RNA followed by reverse transcription and PCR amplification [140] or by addition of a poly(A) tail to footprint RNA followed by reverse transcription with oligo(dT) primers containing adaptor sequences [141]. Traditionally, Ribo-seq library preparation is a time- and resource-intensive process and requires large amounts of input material [142]. Recent methodological advances, such as using bead-based purification to replace column centrifugation [143,144] and using ordered two-template relay (O2TR) to replace inefficient RNA-adapter ligation [145,146], have made it possible to generate high-quality Ribo-seq data with low input material and shorter preparation time.

In human cancers, Ribo-seq data have highlighted widespread translation activities in regions previously annotated as non-coding. Ribo-seq has helped identify noncanonical antigens from many sources of noncanonical ORFs such as 5′ uORFs, 3′ dORFs, and lncRNAs [147], and across cancer types including lymphoma/leukemias [147], CRC [147] melanoma [147,148], and HCC [71]. Nevertheless, as a relatively new technology, Ribo-seq exhibits varying sensitivity and specificity in detecting different types of noncanonical antigens [149]. The most robust discoveries combine Ribo-seq with complementary approaches [148,150,151]. In fact, several cancer-focused databases have been developed by integrating Ribo-seq data in conjunction with other data types. For example, nuORFdb [152], a dedicated database of novel or unannotated ORFs in healthy and cancer samples, was developed with Ribo-seq and mass spectrometry immunopeptidomics data [147]. riboCIRC [153] integrates Ribo-seq and circular RNA sequencing data and catalogs translatable circRNA from various cancers [154]. lncExplore, on the other hand, annotates lncRNA sequences on RNA-seq datasets from human cancers with insights generated from Ribo-seq [155]. Thus, in addition to continued technical optimization, integrating Ribo-seq with complementary methodologies will be important for cross-validation for noncanonical antigen discovery.

### 5.2. Mass Spectrometry (MS): Direct Analysis of the Tumor Immunopeptidome

For the NGS-based strategies discussed above where candidate TSAs are identified based on sequencing information, binding affinity with a patient’s unique repertoire of HLA molecules is predicted as a surrogate for immunogenicity. Most current binding affinity prediction algorithms are trained on datasets derived from in vitro HLA-peptide binding assays. Although these binding predictions are based on large databases of peptides, they remain imperfect for several reasons. First, the in vitro binding affinity data do not fully capture the complexities of endogenous antigen processing and presentation [156]. Furthermore, the training datasets can be skewed, as the peptide ligands are usually selected based on a hypothesis or prior knowledge, and the HLA alleles are biased towards the most-studied, high-frequency alleles in the Caucasian population [157]. Studying the tumor immunopeptidome directly with MS, on the other hand, provides a direct assessment of HLA-bound peptides on the surface of tumor cells. Since its pioneering in the early 1990s [158], MS has been contributing to HLA immunopeptidome profiling and neoantigen discovery in cancer cells [159,160]. Liquid chromatography-tandem mass spectrometry (LC-MS/MS) further increases the analysis power by providing three-dimensional data: chromatographic retention time, *m*/*z* ratio of precursor ions, and *m*/*z* ratio of daughter ions, allowing for peptide characterization at higher resolution [156].

Immunopeptidomic analyses by MS generally adopt either a targeted or discovery-based approach. Whereas targeted MS is designed to validate and quantify known peptides, discovery-based MS enables the unbiased identification of HLA-presented peptides and is therefore the method of choice in TSA discovery. Discovery-based immunopeptidomics can be broadly divided into two categories: data-dependent acquisition (DDA) and data-independent acquisition (DIA) [161]. In DDA, the most abundant precursor ions in MS1 are selected in real-time for sequential MS2 fragmentation. The resulting spectra contain cleaner, individual fragmentation patterns, allowing for direct database searching [162]. DDA thus enables confident identification but can be affected by abundance bias and therefore has less reproducibility between samples. On the other hand, DIA selects all precursor ions within a predefined *m*/*z* window in MS1 for simultaneous MS2 fragmentation, leading to less biased representation and enhanced reproducibility. However, DIA generates highly multiplexed, overlapping fragment ion spectra due to the simultaneous fragmentation of many precursor ions within wide isolation windows. Data analysis typically relies on matching observed spectra to reference spectral libraries. In cancer neoantigen discovery, these libraries can be existing databases, constructed in silico with NGS data, and/or generated by concurrent DDA analysis [163].

To date, MS has helped reveal many noncanonical antigens, including aberrant pre-mRNA splicing [164], lncRNA [77], and PTM [82]. The dependence on existing databases and spectral libraries, however, can lead to challenges in identifying peri-translational TSAs not reflected at the sequencing level. One method to address such limitations is de novo sequencing, where amino acids are directly “sequenced” from fragment ions on the MS spectra using the mass difference between spectral peaks [165]. De novo sequencing has been integrated into several neoantigen discovery workflows that have identified noncanonical antigens, including noncanonical ORFs and PSPs [90,166]. It is important to note that *de novo* sequencing cannot differentiate between amino acids with the same molecular weight, such as leucine and isoleucine, and also assumes fragmentation at every peptide bond, a scenario that is rarely guaranteed. Therefore, accurate false discovery rate (FDR) control remains a significant challenge, necessitating technical and biological validation of assigned peptide sequences [93].

With advances in machine learning and artificial intelligence, MS analysis workflows have undergone successive rounds of optimization. Hybrid approaches integrating DDA, DIA, and de novo sequencing have now been developed and led to streamlined workflows and more effective FDR control [167,168,169,170]. These advances in bioinformatics tools are accompanied by evolving mass spectrometry techniques such as trapped ion mobility spectrometry and high-field asymmetric waveform ion mobility spectrometry [171,172], enabling tumor immunopeptidome analysis from small samples, including dissected clinical specimens or single-cell experiments [173]. This increases the feasibility of integrating MS into neoantigen discovery pipelines in the clinical setting where tumor tissue weight is frequently limited.

### 5.3. Proteogenomics, Where the Ends Meet

**Figure 3:**
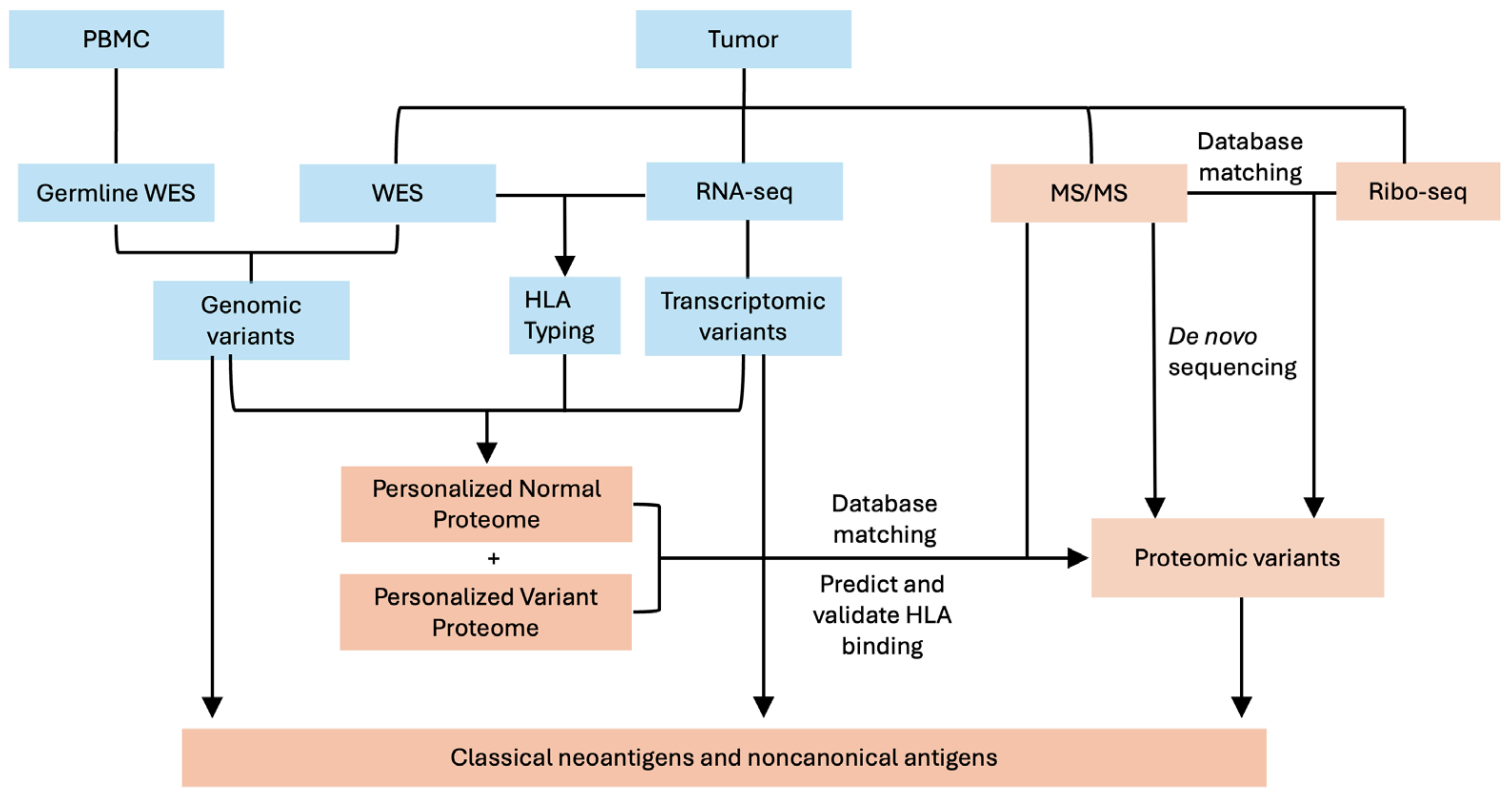
Proteogenomics pipeline.

First coined by Jaffe, Berg and Church [174] in 2004, the term “proteogenomics” now describes the multidisciplinary research field that integrates MS-based proteomics with NGS-based genomics, transcriptomics and translatomics [175] (Figure 3). In cancer neoantigen discovery, proteogenomics (arguably better termed “immunopeptidogenomics” [176]) offers the unique advantage of combining in silico prediction and prioritization of sequencing data with experimental validation through MS. Proteogenomic pipelines have enabled identification of both canonical and noncanonical peptides [150,177,178,179,180], including those arising from non-coding region mutations, PTMs, and aberrant pre-mRNA splicing, which might have been missed by genomic sequencing alone or might not have been prioritized.

The application of proteogenomics pipelines in cancer research (Figure 3) starts with concurrent sequencing and LC/MS-MS analysis of biopsied or resected tumor tissue. Similar to the current workflow for classical neoantigens, DNA- and some RNA-level variants can be derived from WES and RNA-seq, respectively, and validated with MS. Concurrent WES on PBMCs is performed to distinguish between germline and tumor-specific variants, and the patient’s HLA type can be determined with WES and RNA-seq data. The integration of long-read sequencing on both DNA and RNA then enables the discovery of noncanonical sources of TSA such as TEs, aberrant pre-mRNA splicing, and lncRNA. Importantly, both short- and long-read DNA and RNA data can be used to construct a reference, “variant” proteome, which, combined with a publicly available reference normal proteome (such as UniProt [181]), forms the basis of MS database search and spectral library matching. More recent pipelines have incorporated Ribo-seq into the MS search database and have aided in the discovery of noncanonical ORFs [182,183]. Ribo-seq further has the advantage of exhibiting a characteristic triplet periodicity pattern, allowing one to infer the position of ribosomes on the transcript and therefore deduce the correct reading frame [184]. Compared to three-frame translation using traditional RNA-seq data to construct the MS reference database, this effectively shrinks the search space three-fold and can decrease the frequency of false-positive hits.

The predicted TSAs are then prioritized based on their likelihood of being immunogenic and clinically relevant. Important prioritization factors include HLA binding affinity compared to the wild-type counterpart; position of mutation within the HLA-bound peptide, such as anchor vs. non-anchor positions [11]; and biological context, such as driver vs. passenger mutations. Importantly, in proteogenomics, MS data also allows for empirical confirmation of actual peptide-HLA binding and presentation on tumor cells [150,185,186], and thus antigens validated on MS should be prioritized. Rapid developments in proteogenomic workflows and data accumulation across cancer types will likely provide continued insights into the relative contribution and significance of different TSA classes in the tumor immunopeptidome.

## 6. Clinical Relevance of Noncanonical Antigens in Personalized Cancer Vaccine Design

Over the last few decades of cancer vaccine development, considerable efforts have focused on optimizing vaccine design and enhancing immunogenicity. During the tumor-associated antigen (TAA) era, approaches such as altered peptide ligands (APLs) and mimotopes were developed to enhance T cell recognition by engineering naturally occurring tumor antigens. This concept has persisted into the current NGS-based neoantigen era, with ongoing efforts to engineer neoantigen peptide sequences to maximize immune responses [187,188]. The incorporation of immune adjuvants and combination therapies has likewise been pursued to promote antigen presentation and amplify vaccine-induced immune responses.

In parallel, expanding the breadth of intrinsically immunogenic TSAs represented in vaccines provides another potential avenue for improving cancer vaccine efficacy. In light of accumulating evidence that noncanonical antigens constitute a substantial fraction of the tumor immunopeptidome [2,3], can be shared among patients [4,5,6], and may exhibit enhanced immunogenicity [102], together with advances in technologies for their identification, there has been growing interest in systematically characterizing noncanonical antigens and incorporating them into PCV design. Table 2 highlights key clinical and recent preclinical evidence of PCVs containing various classes of TSAs.

A few caveats exist. First, it is worth noting that most efforts on noncanonical antigens remain in the preclinical stage, with only a few exceptions. Although many studies have confirmed the presence of noncanonical antigens in various tumors and tested their immunogenicity in vitro, far fewer have actually administered the noncanonical antigen in the form of a vaccine in vivo in human or animal models. Additionally, there have been no preclinical or clinical studies using experimentally identified PSP-derived TSA as a vaccine epitope. The closest approximation was attempted by Willimsky et al. in 2021 [198], in which the authors generated TCRs against computationally predicted KRAS^G12V- and RAC2^P29L-derived neo-splicetopes and showed TCR recognition of the neo-splicetopes in vitro. However, there was no neo-splicetope-specific T cell response in vivo, and no evidence of their natural processing and presentation was found. Lastly, the body of work on DRiP-derived TSA vaccines centers around the concept of DRiP-containing blebs, or the “DRibble” platform, which captures DRiPs in bulk within autophagosome vesicles and delivers them as a whole tumor cell or multi-antigen vaccine. This contrasts with the classical PCV design workflow, where individual TSAs are identified and characterized.

Several challenges must be addressed before noncanonical antigens can be routinely incorporated into cancer vaccine design in the clinical setting. First, the identification of some classes of noncanonical antigens remains costly, time-consuming, and dependent on high-quality input material. For instance, long-read sequencing can cost around $2000 per genome, while traditional Ribo-seq library preparation takes up to 7 days [199]. These approaches may also be difficult to apply to clinically archived, FFPE tissues, which can contain degraded or limited amounts of nucleic acids. Similarly, although advances in MS have improved sensitivity, immunopeptidomic analyses may require substantial amounts of tumor tissue, with some workflows requiring approximately 100 mg of input material [172]. This may be challenging for small biopsy specimens or surgical samples with low tumor content. A second challenge is the lack of appropriate normal-tissue references for many noncanonical antigen discovery approaches. Unlike WES-based classical neoantigen identification workflows, which typically use matched patient PBMC to distinguish somatic from germline variants, it can be difficult to establish whether a noncanonical antigen is truly tumor-specific or is also expressed in normal tissues but just absent from current reference annotations. Establishing tumor specificity will therefore require comprehensive profiling of normal tissues matching the origin of the tumor, an approach that may be invasive and difficult to implement in clinical practice, as well as improved reference databases.

As proteogenomic pipelines incorporate data from WES, RNA-seq, long-read sequencing, and MS, they may allow for simultaneous discovery of classical neoantigens and noncanonical antigens to better represent the heterogeneous tumor mutational landscape. This can drive concomitant developments in TSA prioritization algorithms, in which different classes of TSAs can be included in a PCV based on their predicted immunogenicity [150,185,186]. Importantly, as alluded to previously in Section 5.2, most current binding/immunogenicity prediction models, such as NetMHCpan, MHCflurry, MHCnuggets, and DeepHLApan, among others, are trained on canonical peptides. Noncanonical antigens, on the other hand, may harbor unique sequence or structural features that current algorithms do not fully capture [200]. Furthermore, noncanonical antigens introduce an additional dimension—antigen class/origin—into the parameters of prioritization. Future TSA prioritization algorithms could integrate information on the TSA’s origin in estimating immunogenicity. For example, TSAs with DRiP/SLiP-like properties may warrant prioritization due to their increased frequency of HLA presentation; TE-derived TSAs may represent highly promising candidates due to the associated viral mimicry state; and TSAs lacking a wild-type counterpart, such as circRNA and other noncanonical ORF-derived peptides, possess higher immunogenicity as they are more likely absent during thymic selection. Importantly, computational frameworks must be supported by continued experimental validation where classical neoantigens and noncanonical antigens are administered as a hybrid vaccine in in vivo studies, as systematic laboratory data are needed to better define the immunogenic potential of distinct TSA classes.

## 7. Conclusions

Expanding the TSA landscape beyond classical, mutation-derived neoantigens has revealed a diverse repertoire of noncanonical antigens with important implications for cancer immunotherapy. Technological advances in immunopeptidomics, ribosome profiling, and proteogenomic approaches are progressively enabling the identification of these previously hidden targets. Although significant challenges remain, including standardization of discovery pipelines, development of noncanonical-focused bioinformatic tools, and translation into clinically scalable platforms, accumulating evidence suggests that noncanonical antigens may represent a substantial and therapeutically relevant component of the tumor immunopeptidome. Incorporating these antigens into next-generation PCVs therefore has the potential to broaden targetable repertoires, improve vaccine efficacy, and expand the applicability of cancer vaccination.

## Figures and Tables

**Table 1:** Source of noncanonical antigens.

Source of TSA	Origin	Event	Identification Strategy
Canonical	Genomic	nsSNV ^1^; INDEL; Fusion gene	WES, RNA-seq
Noncanonical	Genomic/Epigenomic	TE, including ERV	Long-read sequencing (DNA & RNA)
	Transcriptomic	Aberrant splicing; CircRNA	CircRNA sequencing; long-read RNA-seq; Ribo-seq; MS
	Translational	Noncanonical ORF; lncRNA	Ribo-seq; MS
	Post-translational	PTM; PSP	MS
	Antigen Processing	DRiP/SLiP	MS

^1^ Abbreviations: nsSNV: non-synonymous single nucleotide variant; INDEL: insertion/deletion; TE: transposable element; ERV: endogenous retrovirus; circRNA: circular RNA; ORF: open reading frame; lncRNA: long non-coding RNA; PTM: post-translational modification; PSP: proteasome spliced peptide; DRiP: defective ribosomal product; SLiP: short-lived protein; WES: whole-exome sequencing; RNA-seq: RNA sequencing; Ribo-seq: ribosome profiling, MS: mass spectrometry.

**Table 2:** Summary of clinical studies or key recent preclinical studies where noncanonical antigens were used as vaccine epitopes in human or animal models.

Source of Antigen	Preclinical or Clinical	Cancer Type	Key Findings	Reference
TE/ERV	Preclinical	Multiple tumor types including colon, head and neck, bladder, lung, breast, endometrial, etc.	Adenovirus-based cancer vaccines targeting ERVMER34-1 mediated tumor control in colon and breast cancer mouse models expressing ERVMER34-1. When combined with checkpoint blockade, the vaccine promoted expansion of tumor-specific antigen-reactive T cells, which was associated with tumor control in the breast cancer (EMT6) model.	[57]
	Preclinical	Triple negative breast cancer	Adenovirus-based vaccine targeting a novel ERV antigen showed tumor growth inhibition in vaccinated mouse breast cancer models (4T1); splenocyte restimulation with the ERV antigen showed measurable IFNγ production in vaccinated mice.	[189]
Aberrant pre-mRNA splicing	Preclinical	HCC	mRNA vaccine encoding aberrant splicing-derived antigens administered to mice bearing patient-derived xenograft HCC models produced significant tumor regression and enhanced intratumoral infiltration of neoantigen-reactive T cells.	[60]
	Preclinical	CRC	Twenty-two transcript variant-derived antigens were encoded in an mRNA lipid nanoparticle vaccine administered to colon cancer (MC38) mouse models. The vaccine curbed tumor progression, induced robust T cell response, and modulated the tumor microenvironment toward an antitumor profile.	[190]
	Preclinical	CRC	Six splice-derived neoantigens were induced by a pharmacological compound. Subsequent peptide vaccination with these antigens suppressed tumor growth in vivo in colon cancer (MC38) in mouse models.	[191]
CircRNA	Preclinical	Breast cancer and melanoma	Peptide vaccination of a circRNA-encoded peptide induced enhanced infiltration of antigen-specific cytotoxic T cells and led to effective tumor control in melanoma (B16F10) and breast (4T1) mouse tumor models.	[66]
LncRNA	Preclinical	CRC	Both dendritic cell and adenoviral vector vaccines encoding lncRNA-derived antigens drove potent antigen-specific CD8+ T cell responses and inhibited tumor progression in mouse colon cancer (CT26) models.	[78]
	Preclinical	HCC	Two translated peptides from noncanonical ORF within lncRNA in HCC cohorts triggered robust ELISpot response in humanized mice receiving peptide vaccines. No tumor challenge study was conducted.	[71]
PTM	Clinical	Melanoma (NCT01846143)	This Phase I trial vaccinated 15 patients who had undergone surgical resection of melanoma with phosphopeptide pBCAR3, or pIRS2, or both. No Grade 3–4 events occurred. T-cell responses were induced to pIRS2 in 5/12 patients and to pBCAR3 in 2/12 patients.	[192]
	Clinical (ongoing)	Multiple tumor types including head and neck squamous cell carcinoma, triple negative breast cancer, renal cell carcinoma, and high grade serous ovarian carcinoma (NCT05329532)	In this Phase I/II trial, eligible patients received the Modi-1 vaccine, which comprises three citrullinated, adjuvanted peptides targeting vimentin and enolase, aiming to induce CD4+ T cell responses. No serious adverse events were observed. In 13 response-evaluable patients, best overall responses included 1 with partial response, 6 with stable disease, and 6 with progressive disease.	[193]
DRiP/DRibble	Clinical	Non-small cell lung cancer (NCT00850785)	Four patients received autologous Dribbles generated from pleural effusion tumor cells. All 4 patients developed specific antibody responses against potential tumor antigens. 2 of 3 evaluable patients showed autologous tumor-specific immune responses (IL-5, IFNγ, TNFα, IL-10). No clinical efficacy observed in the small cohort: all patients progressed after ≤4 vaccine doses and died.	[194]
	Preclinical	CRC	DRibble-loaded dendritic cell vaccines inhibited tumor growth, prolonged survival, and induced antigen-specific T cell responses more effectively than tumor lysate-loaded dendritic cells in colon cancer (CT26) mouse models.	[195]
	Preclinical	Melanoma, breast cancer	Vaccination of melanoma (B16F10) and breast cancer (4T1) mouse models with DRibble and immune adjuvants inhibited tumor growth and prolonged survival.	[196]
	Preclinical	CRC, breast cancer	A biomimetic autophagosome-based nanovaccine (engineered “DRibble”) containing tumor-specific antigens was able to eradicate 71.4% of established murine colon tumors (MC38) and achieved a tumor inhibition rate of 100% in tumor rechallenge mouse models. When combined with immune checkpoint blockade, the vaccine resulted in delayed tumor growth and 33.3% tumor eradication in a mouse breast cancer (4T1) model.	[197]

## Data Availability

No new data were created or analyzed in this study. Data sharing is not applicable to this article.

## Abbreviations

The following abbreviations are used in this manuscript:

APL	Altered peptide ligand
BSJ	Backsplice junction
circRNA	Circular RNA
CRC	Colorectal cancer
DDA	Data-dependent acquisition
DIA	Data-independent acquisition
dORF	Downstream ORF
DRibble	DRiP-containing bleb
DRiP	Defective ribosomal product
ERV	Endogenous retrovirus
FDR	False discovery rate
HCC	Hepatocellular carcinoma
HLA-I	Human leukocyte antigen class I
HLA-II	Human leukocyte antigen class II
HLA	Human leukocyte antigen
IFNγ	Interferon-γ
IMCR-seq	Induro-RT mediated circRNA-sequencing
INDEL	Insertion/decision
IRES	Internal ribosomal entry site
LC-MS/MS	Liquid chromatography-tandem mass spectrometry
LINE1	Long interspersed nuclear element-1
lncRNA	Long noncoding RNA
MHC	Major histocompatibility complex
MS	Mass spectrometry
MSI	Microsatellite instability
NGS	Next-generation sequencing
nsSNV	Nonsynonymous single nucleotide variant
O2TR	Ordered two-template relay
ONT	Oxford Nanopore Technologies
ORF	Open reading frame
PacBio	Pacific Biosciences
PBMC	Peripheral blood mononuclear cells
PCV	Personalized cancer vaccines
PDAC	Pancreatic ductal adenocarcinoma
PRMT5	Protein arginine methyltransferase 5
PSP	Proteasome-spliced peptide
PTM	Post-translational modification
Ribo-seq	Ribosome profiling
RIN	RNA integrity number
RNA-seq	RNA sequencing
SLiP	Short-lived protein
SMRT	Single-molecule real-time
TAA	Tumor-associated antigen
TCGA	The Cancer Genome Atlas
TCR	T cell receptor
TE	Transposable element
TSA	Tumor-specific antigens
uORF	Upstream ORF
W > F	Tryptophan-to-phenylalanine
WES	Whole-exome sequencing
